# Prolactin Receptor Signaling Is Essential for Perinatal Brown Adipocyte Function: A Role for Insulin-like Growth Factor-2

**DOI:** 10.1371/journal.pone.0001535

**Published:** 2008-02-06

**Authors:** Say Viengchareun, Nathalie Servel, Bruno Fève, Michael Freemark, Marc Lombès, Nadine Binart

**Affiliations:** 1 Inserm, U693, Le Kremlin-Bicêtre, France; 2 Faculté de Médecine Paris-Sud, Université Paris-Sud, UMR-S693, Le Kremlin-Bicêtre, France; 3 Inserm, U845, Paris, France; 4 Faculté de Médecine René Descartes, Université Paris-Descartes, Site Necker, UMR-S845, Paris, France; 5 Division of Pediatric Endocrinology and Diabetes, Duke University Medical Center, Durham, North Carolina, United States of America; Katholieke Universiteit Leuven, Belgium

## Abstract

**Background:**

The lactogenic hormones prolactin (PRL) and placental lactogens (PL) play central roles in reproduction and mammary development. Their actions are mediated via binding to PRL receptor (PRLR), highly expressed in brown adipose tissue (BAT), yet their impact on adipocyte function and metabolism remains unclear.

**Methodology/Principal Findings:**

PRLR knockout (KO) newborn mice were phenotypically characterized in terms of thermoregulation and their BAT differentiation assayed for gene expression studies. Derived brown preadipocyte cell lines were established to evaluate the molecular mechanisms involved in PRL signaling on BAT function. Here, we report that newborn mice lacking PRLR have hypotrophic BAT depots that express low levels of adipocyte nuclear receptor PPARγ2, its coactivator PGC-1α, uncoupling protein 1 (UCP1) and the β3 adrenoceptor, reducing mouse viability during cold challenge. Immortalized PRLR KO preadipocytes fail to undergo differentiation into mature adipocytes, a defect reversed by reintroduction of PRLR. That the effects of the lactogens in BAT are at least partly mediated by Insulin-like Growth Factor-2 (IGF-2) is supported by: i) a striking reduction in BAT IGF-2 expression in PRLR KO mice and in PRLR-deficient preadipocytes; ii) induction of cellular IGF-2 expression by PRL through JAK2/STAT5 pathway activation; and iii) reversal of defective differentiation in PRLR KO cells by exogenous IGF-2.

**Conclusions:**

Our findings demonstrate that the lactogens act in concert with IGF-2 to control brown adipocyte differentiation and growth. Given the prominent role of brown adipose tissue during the perinatal period, our results identified prolactin receptor signaling as a major player and a potential therapeutic target in protecting newborn mammals against hypothermia.

## Introduction

The lactogenic hormones of the placenta (placental lactogens, PL) and pituitary gland (prolactin, PRL) regulate a variety of physiological processes involved in gonadal function and reproduction, mammary development and lactation, adrenal and pancreatic hormone secretion, and intermediary metabolism [1]. In humans and rodents, these actions are mediated via binding of lactogens to the prolactin receptor (PRLR), a class I cytokine receptor linked to activation of the JAK/STAT signaling pathway [2].

In postnatal rodents and ungulates, the PRLR and associated STAT5 activity are detected in most tissues at various stages of development [2], [3]; in the fetus, on the other hand, PRLR emerge during mid-late gestation [4]–[6], when high expression levels are detected in the liver, lung, gastrointestinal tract, olfactory epithelium, renal tubules, pancreatic beta cells, and brown adipose tissue (BAT). The latter is of particular interest because BAT mediates adaptive (nonshivering) thermogenesis [7], the process of heat generation required for thermostabilization and survival during delivery and the transition from intrauterine to extrauterine life. The key effector of adaptive thermogenesis is uncoupling protein 1 (UCP1), whose expression is restricted to BAT mitochondria [8]. UCP1 dissipates the proton gradient at the inner mitochondrial membrane to prevent oxidative phosphorylation, generating heat rather than ATP. A critical upstream regulator of adaptive thermogenesis is peroxisome proliferator gamma co-activator 1 alpha (PGC-1α), which promotes mitochondrial biogenesis [9].

Recent studies have elucidated the molecular mechanisms controlling BAT development. The key transcriptional regulator of adipogenesis in brown, as in white, adipose tissue is PPARγ [10]. Early in the course of adipocyte differentiation, C/EBPβ and δ are expressed transiently and contribute to induction of PPARγ and C/EBPα, which is required to maintain PPARγ expression. C/EBPα also functions synergistically with PPARγ to promote the expression of genes found in both BAT and WAT [11] as well as PGC-1α and UCP1, which are preferentially or exclusively expressed in brown adipocytes.

The expression of BAT genes is regulated by a number of hormones and growth factors including the thyroid hormones, catecholamines, leptin, insulin, and insulin-like growth factor-1 (IGF-1). However, cold adaptation can be maintained in the absence of thyroid hormones, and deletion of thyroid hormone receptor α and β [12] or of β1, β2 and β3 adrenergic receptor subtypes [13], [14] has no effect on BAT PGC-1α mRNA or UCP1 protein levels under non-stressed conditions. Moreover, targeted deletion of the thyroid hormone receptors, the insulin receptor, or insulin receptor substrates 1, 2, 3 or 4, which mediate many of the metabolic effects of insulin and IGF-1, have no effects on BAT mass in newborn mice [12], [15]–[17]. These findings suggest that other hormones or growth factors might contribute to BAT growth and function during the fetal or perinatal periods.

Given their presence in fetal and postnatal plasma and their binding to brown adipocytes *in vivo* and *in vitro* [4], [5], [18], we hypothesized that the lactogenic hormones may play a physiological role in the regulation of BAT development and thermogenesis during the perinatal period. To test that hypothesis, we employed three novel experimental models of lactogen action. The PRLR knockout (KO) mouse [19], which cannot respond to either PL (in utero) or PRL (perinatal and postnatal periods), was used to assess the effects of lactogen resistance *in vivo* on BAT mass, mitochondrial structure, gene expression, and the adaptation to cold. These studies were performed on the day of birth to establish their physiological relevance. Immortalized preadipocyte cell lines derived from the interscapular brown adipose depots of PRLR KO mice were employed to assess the direct effects of PRL signaling on adipocyte differentiation and function *in vitro* and to determine if reintroduction of PRLR can restore brown adipocyte commitment. Finally, the PRL-responsive T37i cell line [18], derived from a BAT hibernoma developed in a transgenic mouse [20], was used to assess the molecular mechanisms by which PRLR signaling controls BAT differentiation.

We discovered that absence of PRLR signaling compromised the growth and differentiation of BAT *in vivo* and *in vitro* and reduced plasma concentrations and BAT expression of insulin-like growth factor-2 (IGF-2), which plays a central role in the control of placental and fetal growth [21], [22]. We therefore examined the regulation of IGF-2 mRNA levels by PRL in isolated brown adipocytes and the effects of IGF-2 on brown adipocyte differentiation and growth; we then determined if exogenous IGF-2 could reverse the effects of PRLR-deficiency on BAT development in immortalized preadipocyte cell lines. Our findings suggest that the lactogens act in concert with IGF-2 to control brown adipocyte differentiation and growth.

## Results

### Brown adipose tissue development is altered in PRLR KO mice

**Figure 1:**
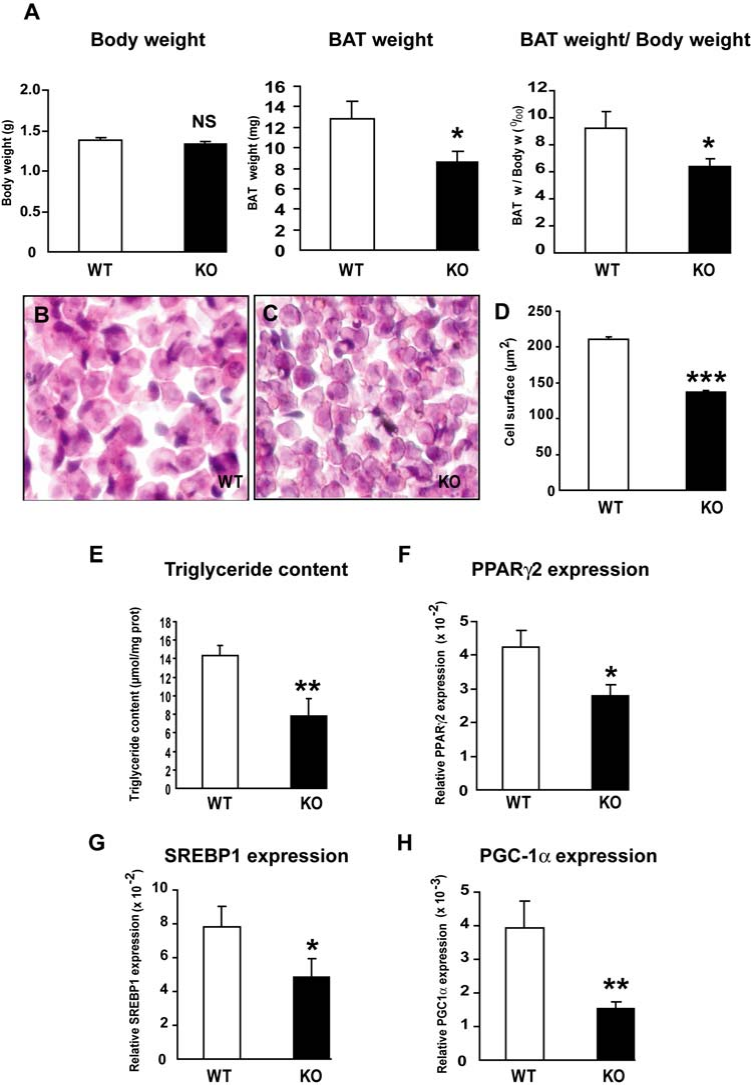
Lack of PRLR impaired brown adipose tissue development in newborns. A) Body weight (BW), BAT weight and BAT/BW ratio were determined in newborn wild type (WT) and PRLR (KO) mice. The BAT weight and BAT/BW ratio were significantly lower in PRLR KO mice (n = 16) than in WT animals (n = 15) (*p<0.05). Results represent mean±SE. B, C and D) Decreased brown adipocyte size in BAT of PRLR KO (C) compared to WT mice (B). BAT of newborn animals were collected, fixed and stained with hematoxylin and eosin. Representative images are presented (objective×60). Adipocyte cell surface was calculated using the Lucia software and a significant decreased was detected in PRLR KO mice (n = 5) *vs* controls (n = 5) (***p<0.001, n = 200 cells /group). E) Intracellular triglyceride content of BAT was significantly reduced in PRLR KO mice (**p<0.01). F, G and H) Drastic decrease of PPARγ2, SREBP1 and PGC-1α mRNA expression in BAT of PRLR KO mice compared to control littermates. BAT of WT and PRLR KO newborn mice were collected, total RNA extracted and processed for quantitative real time PCR analysis of gene expression as described in the Materials and Methods section. Relative expression within a given sample was calculated as the ratio (attomol of specific gene/fmol of 18S). Results are presented as mean±SE of 15–16 animals. * p<0.05 and ** p<0.01.

The demonstration that prolactin receptors (PRLR) are expressed at high levels in brown adipocytes of fetal and newborn rodents [5] and are functional in brown adipocytes [18] led us to explore the physiological relevance of PRL action in BAT *in vivo*. To that end, we characterized BAT development in newborn PRLR KO mice and their wild type (WT) littermates. The body weights of newborn PRLR KO mice were not different from those of WT pups; however the absolute interscapular BAT mass, evaluated by its weight and the BAT/body weight ratio, was significantly reduced in PRLR KO mice as compared with WT littermates (Fig. 1A). These findings suggested that lactogens function as growth factors in BAT during fetal development, since the reduction in PRLR KO BAT mass was detected at birth.

To assess further the impact of PRLR deletion on BAT development *in vivo*, we analyzed the histological appearance of brown adipocytes in newborn PRLR KO and WT mice (Fig. 1B and C). WT brown adipocytes were significantly larger than PRLR KO cells; there was a 35% decrease in mean cell surface of PRLR KO brown adipocytes (Fig. 1D), corresponding to a 50% reduction of cell volume. To determine if the reduction in cell size reflected a decrease in cellular lipids, we compared the triglyceride content of PRLR KO BAT depots with that of WT depots. Triglyceride content was decreased by 40% in the absence of PRLR signaling, highlighting the importance of the lactogens in brown adipocyte function (Fig. 1E). However, the activities of citrate synthase (CS) and of glycerol-3 phosphate dehydrogenase (G3PDH), one of the enzymatic steps of the tricarboxylic acid cycle also implicated in the initiation of lipogenesis, did not differ between WT and KO BAT homogenates (Supplemental data Fig. S1 and Text S1). These observations suggest that the reduction of triglyceride content in PRLR KO BAT results from a defect in adipocyte differentiation rather than a defect in lipogenesis *per se*.

We next examined the expression of adipogenic markers in order to evaluate the impact of PRLR on brown adipocyte differentiation *in vivo*. PPARγ2 as well as SREBP1 expression in BAT were decreased in PRLR KO mice, consistent with a compromised adipocyte differentiation (Fig. 1F and G). In addition, there was a striking reduction in PGC-1α mRNA levels. Since PGC-1α regulates mitochondrial biogenesis and energy production through induction of UCP1 expression and through functional interaction with PPARγ2 and the retinoic acid receptor (RXRα), the thermometabolic capacity of PRLR KO mice was subsequently evaluated.

### Alterations of thermogenesis and BAT mitochondria in PRLR KO animals

**Figure 2:**
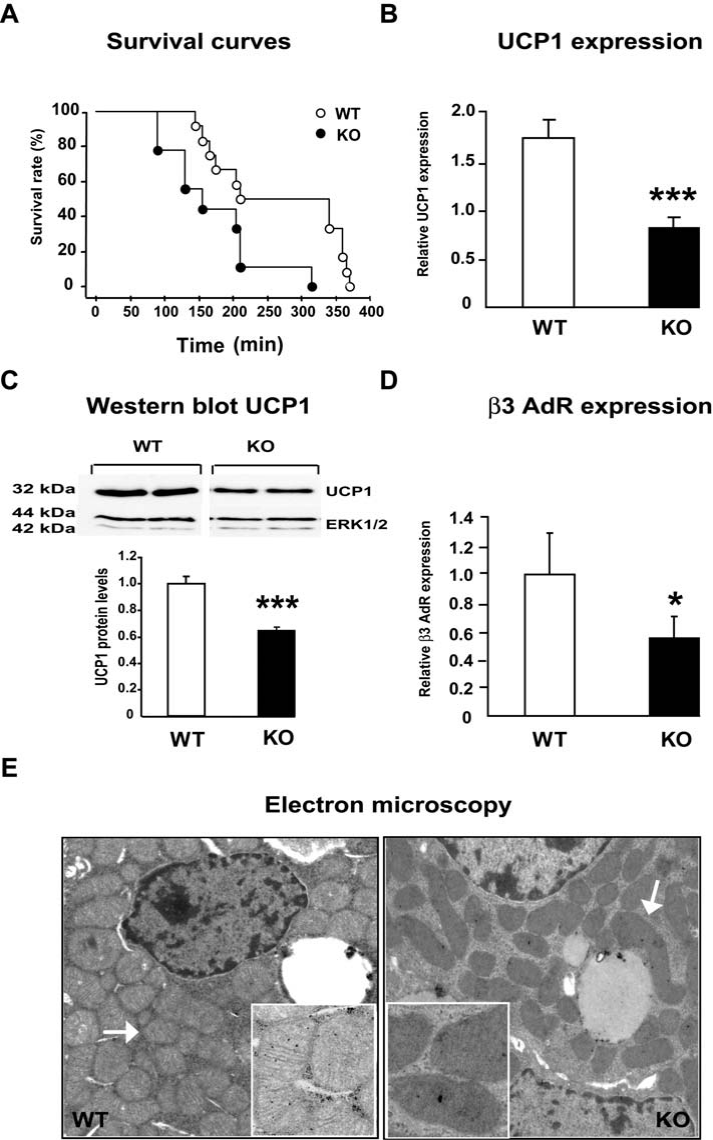
Alteration of thermogenesis in PRLR KO mice. A) PRLR KO newborn mice are more sensitive to cold exposure. Newborn animals were maintained in a 4°C environment and their survival was measured as a function of time (min). Kaplan-Meier representation of survival rate of KO (n = 6) and WT (n = 8) mice reveals a statistical difference (p<0.022). B) UCP1 mRNA expression in BAT of PRLR KO mice was significantly reduced compared to WT littermates. Quantitative real time PCR analysis of UCP1 mRNA expression was performed as described above. Results, expressed as attomol of UCP1/fmol of 18S, represent mean±SE of 15–16 animals (*** *p*<0.001). C) Western blot analysis of UCP1 expression in BAT of PRLR KO compared to WT mice. BAT of newborn animals were collected, lysed and 2 µg of proteins from homogenates were processed for immunoblotting with specific antibodies against UCP1 and ERK1/2. Quantification of UCP1 signal ratio was measured by densitometry using the Quantity One software (Biorad) and a significant decreased was detected in PRLR KO mice vs controls (***p<0.001, n = 6 per group). D) β3 adrenoceptor expression in BAT of PRLR KO mice was significantly reduced compared to WT littermates. Results, expressed as attomol of β3AdR /fmol of 18S, represent mean±SE of 15–16 animals (*p<0.05). E) Representative transmission electron-microscopic images of brown adipocytes of interscapular BAT from a WT newborn mouse and PRLR KO littermate. WT brown adipocytes exhibited intracytoplasmic lipid vacuoles and numerous mitochondria. Note that the mitochondria are extremely dense, compacted and characterized by their round shape and packed cristae encompassing the entire organelles (see arrow). Virtually, no cytoplasmic compartment is visible. In contrast, mitochondria in PRLR KO brown adipocytes are smaller, less packed and presented with elongated and fusiform features. The amount of cristae seems to be reduced (inset). Magnification X 15,000.

To assess the effects of lactogen resistance (PRLR deletion) on thermogenesis, we subjected newborn mice to a cold challenge and calculated their survival rates during the 400-min period, since alternative methods such as measurement of rectal temperature or indirect calorimetry could not be performed on newborn animals. Newborn PRLR KO pups were more sensitive to cold exposure than their WT littermates; 50% mortality was observed at 125 and 200 min, in PRLR KO and WT mice, respectively, and 30% of the WT mice, but none of the PRLR KO mice, survived more than 300 min (Fig. 2A).

The key effector of adaptive thermogenesis and cold adaptation is UCP1. To determine if UCP1 expression is altered in PRLR KO animals, we quantified UCP1 expression in interscapular BAT. Both UCP1 mRNA and protein levels were significantly reduced in BAT of newborn PRLR KO mice (Fig. 2B, 2C), suggesting that UCP1 deficiency accounts for their thermogenic defect. Given the prominent role played by the β3 adrenoceptor (β3Adr) in mediating catecholamines effects on thermogenesis in rodents [23], β3Adr transcripts were quantified in WT and PRLR KO neonates (Fig. 2D); its significant decrease strongly supports the thermogenic defect, and therefore the physiological relevance of PRLR signaling BAT function.

BAT differs from white fat by its high degree of vascularization and mitochondrial density. Electron microscopic analysis of BAT demonstrated striking differences in mitochondrial morphology of PRLR KO mice compared to WT littermates (Fig. 2E). Smaller, sparse and often elongated mitochondria were present in the cytoplasm of PRLR KO brown adipocytes. This was associated with reduced fat droplets, in accordance with morphological and functional BAT abnormalities. Of note, the number of mitochondrial DNA copies in brown adipocytes of PRLR KO mice did not differ from those in WT mice (supplemental data Fig. S2A). Accordingly, cytochrome c oxidase (COX) activity, the fourth complex of the respiratory chain selected as representative of the mtDNA-dependent mitochondrial activities, did not vary between KO and WT BAT (supplemental data Fig. S2B and Text S1). Thus, the respiratory chain activity of lactogen-resistant brown adipocytes was preserved while their thermogenic capacity was clearly impaired. Taken together, these observations strongly suggest that lactogen signaling plays a pivotal role in the setting and/or maintenance of thermogenic function in BAT.

### Differentiation of immortalized PRLR deficient preadipocytes is impaired

**Figure 3:**
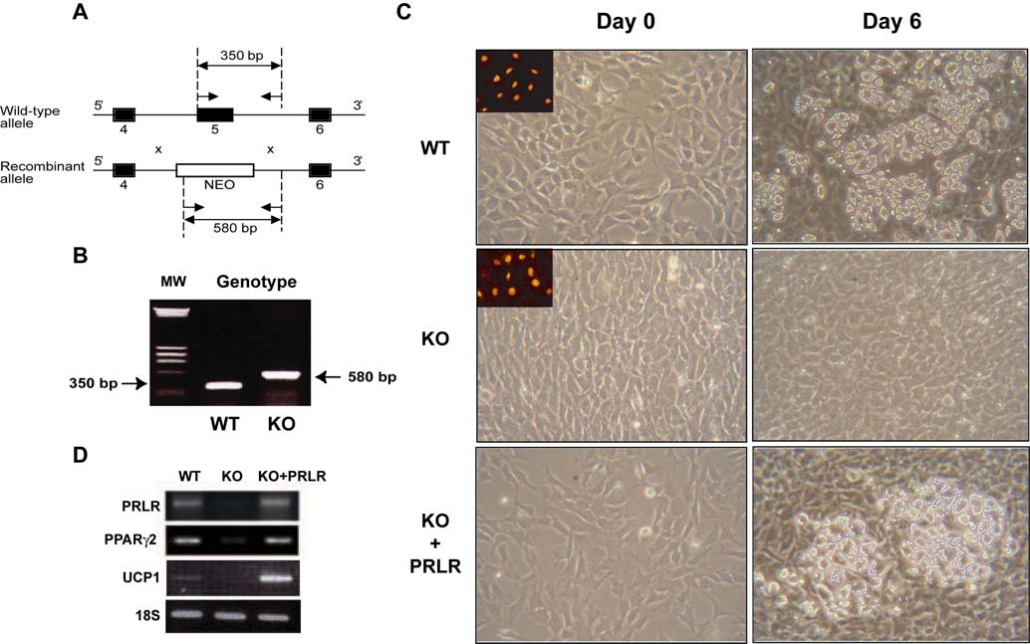
PRLR is crucial for brown adipocyte differentiation. A) Schematic representation of the null mutation of the PRLR gene by homologous recombination and gene targeting techniques. Black boxes represent exon 4, 5 and 6. NEO: neomycin gene ; arrows indicate the position of primers used for PCR. B) PCR genotyping of immortalized brown adipocyte cell lines. C) Morphologic features of wild-type (WT), PRLR KO (KO) and rescued-PRLR (KO+PRLR) brown adipocytes on day 0 and day 6 of the differentiation program. All cell lines presented fibroblast-like morphology (day 0). Inset: immunodetection of the SV40 large T antigen in the nucleus of WT and PRLR KO brown adipocytes. WT, KO and KO+PRLR cell lines were cultured in the presence of 20 nM insulin and 2 nM T3 for 6 days. Light microscopic examination revealed the presence of numerous intracytoplasmic lipid droplets in the WT brown adipocyte cell line whereas PRLR KO brown adipocytes were unable to accumulate lipid droplets in their cytoplasm. Overexpression of PRLR in PRLR KO brown adipocytes (KO+PRLR) by stable transfection of a murine PRLR expression vector restores their ability to differentiate into mature brown adipocytes. D) RT-PCR analyses of PRLR, PPARγ2, UCP1 mRNA expression and 18S RNA used as internal control in WT, KO and rescued-PRLR (KO+PRLR) brown adipocytes. Note the reappearance of a strong PPARγ2 expression in rescued-PRLR differentiated brown adipocytes.

To investigate whether PRLR deficiency limits the ability of brown preadipocytes to undergo adipogenic conversion, we isolated cells from the stromal vascular fraction of interscapular BAT of PRLR KO and WT newborns. Several brown preadipocyte cell lines were established after stable transfection of the P1-TAg expression vector, which contains the immortalizing SV40 large T Antigen (TAg) under the control of the proximal promoter of the human mineralocorticoid receptor gene. This transgene was previously used to generate the T37i brown adipocyte cell line [20] (Fig. 3A and B). The differentiation capacities of PRLR KO and WT cell lines were assessed in order to decipher the molecular events by which PRLR signaling contributes to the commitment to the brown adipose phenotype. Fig. 3C presents results obtained with representative clones of WT and PRLR KO preadipocytes. At post confluence (day 0) both cell lines exhibited fibroblast-like morphology and expressed the SV40 TAg, as demonstrated by its nuclear immunodetection (inset). After 6 days of exposure to insulin and T3, the WT preadipocytes differentiated and accumulated triglycerides within multilocular fat droplets (Fig. 3C, upper panel). In contrast, PRLR KO preadipocytes failed to accumulate cytoplasmic lipid droplets (Fig. 3C, middle panel), suggesting that PRLR signaling plays a crucial role in brown adipocyte differentiation. Similar results were obtained in all other independent cell lines derived from PRLR KO animals (data not shown).

To assess further the role of PRLR signaling in response to PRL and PL which are present in the fetal calf serum, we stably transfected a murine PRLR expression vector into PRLR KO preadipocytes (KO+PRLR) and examined the adipogenic activation cascade. After 6 days exposure to the differentiation mixture (insulin + T3), PRLR-rescued cells accumulated triglycerides and acquired the morphologic appearance of mature brown adipocytes (Fig. 3C, lower panel). RT-PCR analysis performed on WT, PRLR KO and PRLR-rescued cell lines confirmed that expression of PRLR correlated with that of PPARγ2, a marker of adipocyte differentiation, and of UCP1, a marker of brown adipocyte lineage (Fig. 3D). These findings establish conclusively a direct role for lactogen signaling in brown adipocyte differentiation.

### Identification of IGF-2 as a target of lactogen signaling in BAT

**Figure 4:**
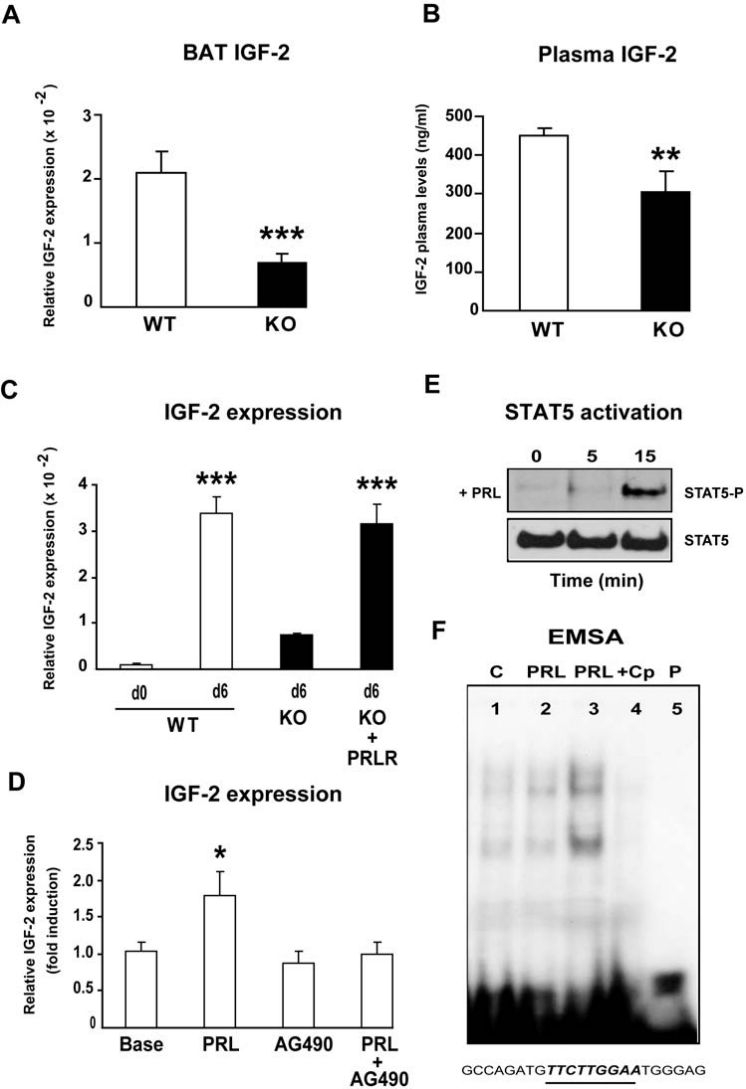
IGF-2 is a target gene of PRL in brown adipose tissue. A) qPCR analysis of IGF-2 mRNA expression of BAT from newborn mice, results are expressed as attomol of IGF-2/fmol of 18S, represent mean±SE of 15–16 animals (*** *p*<0.001). B) Plasma IGF-2 levels. Values in WT (n = 16) and in PRLR KO (n = 21) animals represent the mean±SE. (** *p*<0.01). C) IGF-2 mRNA is expressed as a function of brown adipocyte differentiation. IGF-2 mRNA expression was determined in immortalized WT, PRLR KO, and PRLR-rescued brown adipocytes at day 0 or day 6 of the differentiation program. Results represent mean±SE of three independent experiments performed in duplicate (*** p<0.001 *vs* WT at d0). D) Induction by PRL of IGF-2 mRNA expression in T37i cells. Differentiated T37i cells were incubated overnight with 10% horse serum known to lack prolactin and placental lactogens, and were stimulated or not by PRL (100 ng/ml) for 3 h. AG490 (100 µM) was added 15 min prior to PRL exposure. IGF-2 mRNA expression was expressed as mean±SE of 12 independent experiments (* *p*<0.05). E) STAT5 activation by PRL in T37i cells. Differentiated T37i cells, were stimulated with 100 ng/ml PRL for 5 and 15 min. Protein lysates were subjected to immunoprecipitation with anti-STAT5 antibody. Samples were submitted to a SDS-PAGE and western blotting analysis using anti-phosphotyrosine STAT5 and STAT5 antibodies. F) Specific binding to a STAT response element located in the IGF-2 promoter region. The sequence of STAT-RE is underlined. Nuclear extracts from untreated (8 µg of proteins, lane 1) and PRL-treated T37i cells (8 µg of proteins, lane 2 or 16 µg of proteins, lane 3) were analyzed for binding to a double-stranded STAT-RE oligonucleotide. Specific complexes were identified by competition experiments in which 100 ng unlabeled STAT-RE was added (lane 4). Free probe (lane 5).

BAT differentiation is under the control of many hormones and growth factors including insulin and IGF-1. Since IGF-2 has been previously shown to mediate PRL signaling during mammary gland morphogenesis [24], we hypothesized that this growth factor might be a mediator of PRL-induced adipocyte differentiation. To test this possibility, we first compared the levels of IGF-2 mRNA in BAT of newborn PRLR KO mice with those in WT mice. IGF-2 transcript levels were 70% lower in BAT of PRLR KO mice than in BAT of WT littermates (Fig. 4A), indicating that endogenous IGF-2 gene expression might be under the direct control of PRLR signaling in brown adipocytes. There was also a 35% reduction in IGF-2 plasma levels in PRLR KO neonates (Fig. 4B), providing further evidence that lactogens regulate IGF-2 production during the fetal and perinatal periods.

Support for a role for PRLR signaling in IGF-2 production was provided by studies of the effects of PRL on IGF-2 expression in brown adipocytes *in vitro*. We first examined the changes in IGF-2 mRNA levels during insulin/T3-induced differentiation of immortalized brown preadipocytes from newborn WT mice. As shown in Fig. 4C, IGF-2 mRNA levels were significantly higher in differentiated than in undifferentiated cells; thus brown adipocyte differentiation is accompanied by a sharp induction of IGF-2 expression. We next compared the levels of IGF-2 mRNA in immortalized PRLR KO cells and KO+PRLR cells with those in WT cells. IGF-2 mRNA levels were 75% lower in insulin/T3-treated PRLR-KO cells than in WT cells on day 6. IGF-2 expression was restored by re-introduction of PRLR into PRLR KO cells. Finally, we showed that a 3 h-PRL exposure provokes a 2-fold increase in the levels of IGF-2 mRNA in T37i brown adipocytes (Fig. 4D). Collectively, these findings establish a critical role for lactogen signaling in brown adipocyte IGF-2 expression.

The mechanisms by which lactogens regulate IGF-2 are currently unknown. Since PRL is known to mediate most of its effects through activation of the JAK2/STAT5 pathway, we determined if the specific JAK2 inhibitor AG490 could blunt the effect of PRL on IGF-2 expression in T37i cells. As illustrated in Fig. 4D, we showed that the induction by PRL of IGF-2 mRNA levels was abolished in the presence of AG490. We next showed that PRL treatment of differentiated T37i cells induced phosphorylation of STAT5 within 15 min (Fig. 4E). Since the IGF-2 gene promoter contains multiple consensus STAT binding sites, we performed electromobility shift assays with nuclear extracts of untreated or PRL-treated T37i cells and the STAT response element located −3184 bp upstream of the transcription initiation start site of IGF-2 gene (exon 1) (Fig. 4F). Specific oligonucleotide-protein complexes were detected in PRL-stimulated extracts, a finding compatible with a model in which PRL regulates IGF-2 transcription through STAT5 activation.

### IGF-2 induces brown adipocyte proliferation and differentiation

**Figure 5:**
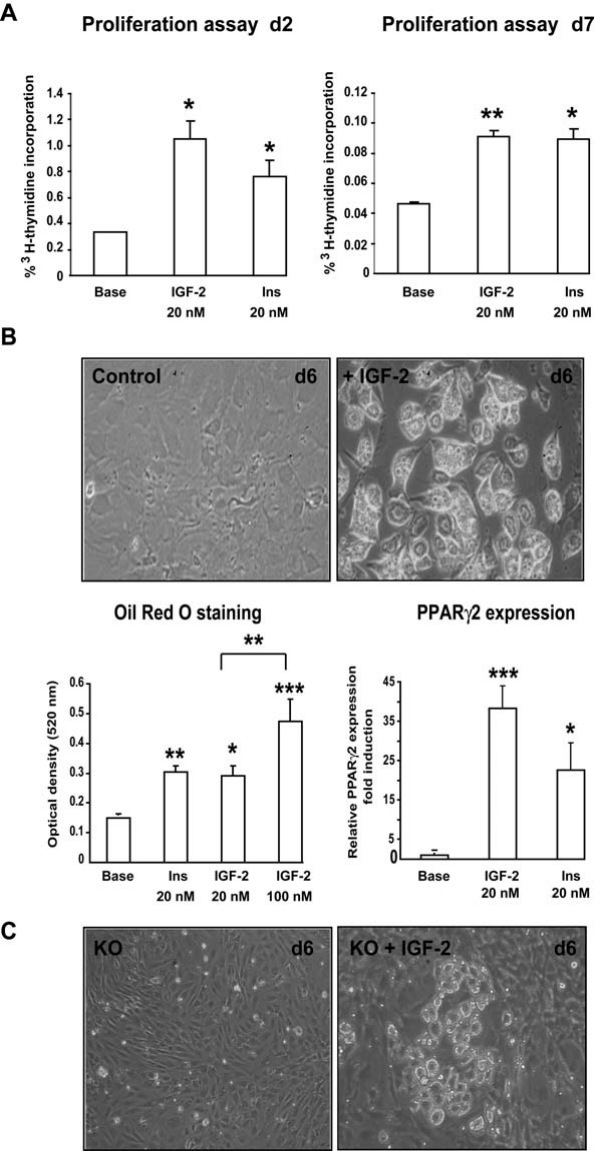
IGF-2 induces brown adipocyte proliferation and differentiation. A) Undifferentiated (d2) and differentiated (d7) T37i cells were treated with 20 nM IGF-2 or 20 nM insulin for 8 h. [^3^H]-thymidine (1 µCi per well) was then added to cells. Incorporation of [^3^H]-thymidine into DNA represents the mean±SE (three independent experiments performed in quadruplicate) Statistical significance: * p<0.05 and ** p< 0.01. B) T37i cells were grown in medium supplemented with 10% dextran-coated charcoal serum in the absence (control) or in the presence of 20 nM IGF-2 for 6 days. A full differentiation switch was observed with IGF-2. (Magnification X 20). Oil Red O staining was performed at d6 in the absence (base) or in the presence of 20 nM IGF-2, 20 nM insulin or 100 nM IGF-2. Oil Red O stained-cells were solubilized in 10% SDS and the OD_520 _measured. Results are mean±SE of three independent experiments. Statistical significance: *p<0.05, **p<0.01 and ***p<0.001, compared to untreated cells. Induction of PPARγ2 mRNA expression in T37i cells treated or not by 20 nM IGF-2 or 20 nM insulin for 7 days. Quantitative real time PCR analysis of PPARγ2 mRNA expression was performed, results expressed as attomol of PPARγ2/fmol of 18S, represent mean±SE of three independent experiments performed in duplicate expressed as fold induction *vs* undifferentiated cells (*p<0.05 and *** *p*<0.001). C) PRLR KO preadipocyte cells were grown in medium supplemented with 10% dextran-coated charcoal serum without or with 20 nM IGF-2 for 6 days where the appearance of fully mature adipocyte foci were observed (Magnification×20).

Given the effects of lactogen signaling on IGF-2 expression in BAT, we addressed the role of IGF-2 in preadipocyte proliferation and adipogenesis. To test whether IGF-2 was able to stimulate mitogenesis of brown adipocytes, we exposed postconfluent (day 2) and differentiated (day 7) T37i cells to 20 nM IGF-2 or insulin (which served as a positive control) and measured ^3^H-thymidine incorporation (Fig. 5A). Treatment of proliferating cells with nanomolar concentrations of IGF-2 induced a 3-fold increase in ^3^H-thymidine incorporation. IGF-2 stimulated a 2-fold increase in ^3^H-thymidine incorporation even in differentiated cells, which as expected had lower basal rates of DNA synthesis than undifferentiated cells. The potency of IGF-2 in these respects was comparable to that of insulin. Longer exposure to IGF-2 stimulation induced morphological changes and the appearance of intracytoplasmic lipid droplets in T37i cells, demonstrating a major impact of IGF-2 on brown adipocyte differentiation (Fig. 5B). Quantification of Oil Red O staining and measurement of PPARγ2 mRNA levels established that IGF-2 was as or more potent than insulin in induction of brown adipocyte differentiation (Fig. 5B).

The mechanisms by which IGF-2 stimulates adipogenesis are currently unknown, but the receptors that mediate most IGF-2 effects in the fetus and placenta, namely insulin receptor form A (IR-A) and IGF-1 receptor (IGF-1R) [25] were expressed in brown adipocytes (Supplemental data Fig. S3 and Text S1). In addition, IGF-2 receptor (IGF-2R), which mediates IGF-2 turnover by endocytosis, was also detected in brown adipocytes. Since IGF-2 and insulin have been shown to differentially regulate gene expression through IR-A [26], the respective contributions of insulin and IGF-2 signaling in brown adipocyte proliferation and differentiation remain to be established.

### IGF-2 can partially reverse the effect of PRLR-deletion on BAT differentiation

Given the adipogenic effects of IGF-2 in T37i cells and the induction by PRL of IGF-2 expression, we next determined if incubation with exogenous IGF-2 could reverse the defect in brown adipocyte differentiation observed in PRLR KO cells. As shown in Fig. 5C, IGF-2 promoted partial differentiation of PRLR KO cells, with appearance of foci of mature adipocytes. This finding suggests that IGF-2 can bypass the absence of PRLR and exert its actions downstream of PRL signaling. Our findings provide strong evidence that IGF-2 constitutes one of the major PRL targets in brown adipocytes and are compatible with an autocrine regulatory loop in which PRL stimulates endogenous IGF-2 production, which in turn facilitates activation of adipogenesis in BAT.

## Discussion

The lactogens play important roles in carbohydrate metabolism through effects on pancreatic beta cell mass and insulin production [27], [28] and peripheral insulin sensitivity [29], however their roles in energy homeostasis and lipid metabolism are poorly understood [30]. In this study, we demonstrate that lactogen signaling is essential for brown adipocyte growth and differentiation, thermogenic gene expression, and the adaptation to cold stress. Newborn PRLR KO mice have impaired BAT development as illustrated by sharp decreases in BAT weight, adipocyte size, and triglyceride content. The impaired development of BAT is underscored by a significant decrease in the expression of UCP1, which could be the consequence of altered BAT development, as illustrated by the decrease of PPARγ2 and its coactivator PGC-1α. It is well established that PPARγ2 is important for the development of BAT since mouse models with adipose-specific PPARγ ablation have reduced or absent interscapular BAT [31]. Maladaptive responses to cold have been described in mouse models with deletions in PGC-1α [32] and PPARγ [33]. Moreover, the cold sensitivity of PRLR KO neonates is also amplified by the reduced expression of β3Adr, a key effector for UCP1 expression in rodents [23]. Importantly, we observed a major thermoregulatory defect in PRLR KO animals at birth, highlighting the physiological importance of PRL signaling in adaptive thermoregulatory responses to cold challenge. Previous studies [34] demonstrating a thermogenic effect of PRL in sheep are consistent with our findings. It is currently unclear if the thermogenic effect of PRL is exerted throughout development, but the body temperature of PRLR KO adult mice was reported significantly lower than in WT animals [35].

To assess the impact of lactogen action on brown adipocyte physiology, we examined the effects of PRLR deletion on brown adipocyte differentiation *in vitro*. We showed that immortalized brown preadipocytes from WT newborn mice differentiated during exposure to insulin and T3 and expressed the adipogenic genes PPARγ2 and UCP1. In striking contrast, PRLR-deficient cells failed to differentiate or to express these adipogenic genes despite long-term treatment with insulin and T3. This finding indicates that PRLR signaling, like insulin/IGF-1 signaling, is required for BAT differentiation. Whether or not lactogen action is also required for white adipose tissue differentiation is unclear, but PRL induces PPARγ and C/EBPβ mRNA levels in NIH 3T3 cells [36] and adult female PRLR KO mice have reductions in abdominal fat mass and decrease in adipocyte numbers [37]–[39]. Τhis illustrates that beyond the role of PRLR signaling in adaptive thermogenesis at birth, this pathway may also affect later, white adipose tissue development and metabolism.

A novel finding of our study is the first demonstration that IGF-2 serves as a downstream target of lactogen action in the differentiation of brown adipocytes. We showed that PRLR deletion is accompanied by reductions in BAT IGF-2 mRNA levels *in vivo* and *in vitro* and that PRL stimulates IGF-2 expression in brown adipocytes. The latter effect appears to be mediated through JAK/STAT induction of IGF-2 transcription. We then showed that IGF-2 stimulates the proliferation and differentiation of brown adipocytes and reverses partially the defects in brown adipogenesis observed in PRLR-deficient cells. Even though IGF-2 only represents one of the molecular PRL targets involved in BAT growth and differentiation, this illustrates that it can bypass the absence of PRLR and thereby acts downstream of lactogen signaling. The molecular mechanisms by which IGF-2 exerts its actions are not entirely clear, but the receptors that mediate its effects on placental and fetal growth, namely, the IGF-1R and IR-A, are expressed in the BAT of newborn mice.

It is of considerable interest that plasma IGF-2 levels were reduced significantly in PRLR KO mice on the day of birth. This new observation suggests that lactogenic hormones that circulate in the fetus in late gestation (in rodents, PL, in humans, PL, PRL, and growth hormone) may also induce IGF-2 expression in tissues other than BAT; in theory, these could include the placenta and fetal liver, adrenal, pancreatic beta cells, and mesenchymal tissues, all of which express PRLR as well as IGF-2 [4]–[6], [21]. In rodents, the expression of IGF-2 ceases a few weeks after birth and postnatal murine growth is IGF-2 independent [21]. In contrast, IGF-2 levels are maintained throughout development in humans, and IGF-2 levels are variably decreased in children with growth hormone deficiency [40].

**Figure 6:**
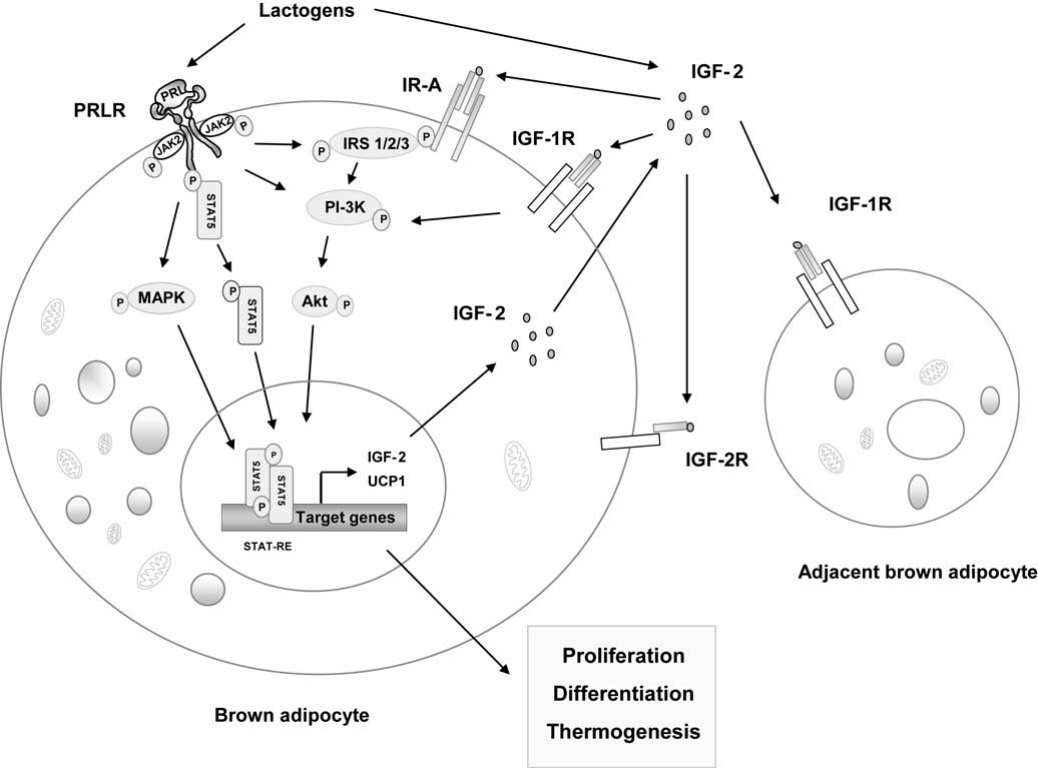
Proposed model of regulation of brown adipocyte function by PRL/JAK2/STAT5 /IGF-2 pathway

The reductions in BAT IGF-2 expression and plasma IGF-2 concentrations in PRLR KO neonates suggest a model whereby lactogen action in BAT may be mediated in part through systemic and/or local IGF-2 production. This model is illustrated in Fig. 6. Prolactin and/or PL bind to the PRLR of the brown adipocyte leading to the activation of various signaling pathways including the MAP kinase (MAPK), phosphatidyl-inositol 3-kinase (PI-3K), and JAK2/STAT5 pathways and potentiation of IRS signaling [18]. This results in transcriptional activation of genes including IGF-2, which is secreted and exerts autocrine/paracrine/endocrine effects by binding to IR-A and IGF-1R. Lactogen signaling thereby promotes cell proliferation, brown adipocyte differentiation and UCP1-mediated thermogenesis.

The finding that IGF-2 reverses only partially the effects of PRLR deletion suggests that PRL target genes other than IGF-2 might be of major pathophysiological importance. Interestingly, direct comparison between the predictive anti-adipogenic genes [41] and the putative PRL target genes identified by microarrays [24] reveals a strikingly homologous list of candidates. They include genes related to cell adhesion and extracellular matrix [42], Wnt signaling pathway [43], cell growth and maintenance which have been already described to play roles in adipocyte biology. Additional experiments must be undertaken to ascertain the involvement of the gene candidates linking PRLR signaling and adipocyte differentiation.

In summary, our findings reveal the critical role played by PRLR signaling in brown adipocyte growth and differentiation, thermogenesis, and the response to cold stress. The mediation of lactogen action by induction of IGF-2 expression provides a novel mechanism subserving perinatal adaptation and energy homeostasis. Given the importance of intrauterine programming and BAT in energy balance, our findings implicate new roles for the lactogens in the interface between fetal and perinatal development and metabolic disease.

## Materials and Methods

### PRLR KO mice

All experimental designs and procedures are in agreement with the guidelines of the animal ethics committee of the Ministère de l'Agriculture (France). WT, and PRLR KO mice on a pure 129 genetic background were generated by heterozygous crossbreeding [19]. PCR analysis of tail DNA determined the genotypes of the offspring as described previously [44].

### Histological analyses

BAT was isolated from the interscapular space of newborn WT and PRLR KO mice, fixed in 4% paraformaldehyde, dehydrated and embedded in paraffin. Sections (4 µm) were deparaffinized in xylene, rehydrated and stained with hematoxylin eosin. The adipocyte surfaces were analyzed using a Lucia software on 200 sections per genotype. Data are mean±SE, for 4 mice per genotype.

### Cell extracts, biochemical determinations, and enzyme assays

BAT was homogenized in 25 mM Tris, pH 7.5, 1 mM EDTA. A fraction of the homogenate was stored at −80°C. The remaining fraction was centrifuged at 10,000×*g* for 10 min at 4°C, and the supernatant was kept at −80°C until use. Aliquots of homogenates and supernatants were used to determine protein content. Triglyceride content was determined on homogenates with PAP150 triglyceride kit (Biomérieux, Marcy L'Etoile, France).

### Electron-microscopic analyses

BAT were washed with PBS and fixed for 4 h with 2% glutaraldehyde (Merck)-2% paraformaldehyde (Sigma) in 0.1 M phosphate buffer, pH 7.4 (Merck) at room temperature. Tissues were rinsed in PBS, postfixed with 2% osmium tetroxyde (Uptima, Montlucon, France) for 30 min, dehydrated in graded ethanol, and embedded in Durcupan (Fluka, St Quentin, France). Ultrathin sections were realized and examined with a JEOL 1010 electron microscope.

### RT-PCR

Total RNA was extracted from cells with TRIZOL reagent (InVitrogen, Cergy Pontoise, France) according to the manufacturer's recommendations and RNA were thereafter processed for RT-PCR, as previously described [45]. Table 1 indicates primer sequences of genes analyzed by RT-PCR or by quantitative real time PCR.

**Table 1:** Primer sequences of genes analyzed in PCR, RT-PCR and Real Time PCR

Name	Accession numbe	Amplicon	Sense primer	Antisense primer
18S	X00686	66 bp	CCCTGCCCTTTGTACACACC	CGATCCGAGGGCCTCACTA
PRLR	BC005555	673 bp	GAGAAAAACACCTATGAATGTC	AGCAGTTCTTCAGACTTGCC
PPARγ2	U09138	161 bp	GCATCAGGCTTCCACTATGGA	AAGGCACTTCTGAAACCGACA
SREBP1	BC056922	152 bp	CGGCTGTTGTCTACCATAAGCTG	CATAGATCTCTGCCAGTGTTGCC
PGC-1α	AF049330	162 bp	CCCAGGCAGTAGATCCTCTTCAA	CCTTTCGTGCTCATAGGCTTCATA
UCP1	BC012701	150 bp	TCACCTTCCCGCTGGACACT	GCAGGCAGACCGCTGTACAG
β3AdR	NM_013462	150 bp	TGCGCACCTTAGGTCTCATTAT	AAGGCGGAGTTGGCATAGC

The abbreviations of the genes, their GENBANK or NCBI accession number, the size of the amplicon and 5′ to 3′nucleotide sequences of the sense and antisense primers are presented.

### Quantitative real time PCR

Specific gene expression was quantified by real time PCR, using an ABI 7300 Sequence Detector (Applied Biosystems, Foster City, CA). Briefly, 1 µg of total RNA was treated using DNase I Amplification Grade procedure (Invitrogen). RNA was thereafter reverse-transcribed with 200 units of reverse transcriptase using the Superscript™ II kit (InVitrogen), then samples were diluted 10-fold and 1/20^th^ of the reverse transcription reaction was used for PCR using the qPCR™ Mastermix Plus for Sybr™ Green I (Eurogentec, Seraing, Belgium), as previously described [45]. Controls without reverse transcriptase and without template were included to verify that fluorescence was not overestimated by residual genomic DNA amplification or from primer dimer formation. Moreover RT-PCR products were analyzed in a post-amplification fusion curve to ensure that a single amplicon was obtained. Quantification was performed by the standard curve method. Standard curves were generated by serial dilutions of a linearized plasmid containing the specific amplicon, spanning six orders of magnitude, yielding a correlation coefficient of at least 0.98 in all experiments. For all experiments, PCR efficiency was close to 1, indicating a doubling of DNA at each PCR cycle, as expected. Ribosomal 18S RNA was used to normalize for RNA quality, quantity and RT-efficiency. Relative expression within a given sample was calculated as the ratio (attomol of specific gene/fmol of 18S). Results are expressed as mean±SE and represent the relative expression compared to that obtained with control animals or cells.

### Kaplan-Meier

All experiments and animal care were performed in accordance with the animal facility of the Faculty of Medicine Necker**.** Cold tolerance was accessed by housing newborn mice at 4°C. When no sign of tactile stimulation was observed, the mice were sacrificed. We found that mice up to 6 h were considered without using death as an end-point.

The survival rate was calculated and expressed according to Kaplan-Meier representation.

### Western blot analyses

Total protein extracts were prepared from BAT isolated from WT and PRLR KO mice. Tissues were washed twice with ice-cold PBS and lysed as described in [18], then 2 µg of total protein were directly submitted to SDS-PAGE. After protein blotting on a nitrocellulose membrane (Biorad, Marnes-La Coquette, France), blots were incubated for 1 h at room temperature in 5% milk-Tris buffer saline/ 0.1% Tween before incubation with the goat anti-UCP1 (1∶3000) (a gift from Pr. D. Ricquier) overnight at 4°C. After extensive washes, membranes were incubated with a goat peroxidase-conjugated second antibody (1∶2000) for 1 h at room temperature and proteins were visualized as mentioned above. After milk saturation, blots were reincubated overnight with rabbit anti-ERK1/2 (1∶1000) (Upstate Biotechnology, Charlottesville, VA) then incubated with a rabbit peroxidase-conjugated second antibody (1∶10000) for 1 h at room temperature and proteins were visualized by use of the ECL^+^ detection kit.

### IGF-2 measurement

IGF-2 was measured by RIA [46] after extraction of IGF binding proteins on a BioGel P-10 (Bio-Rad, Hercules, CA) column equilibrated in 1 M acetic acid. The method has been validated for complete separation of IGFBPs from IGFs in serum and other biological fluids. The IGF-2 assay uses recombinant human IGF-2 as standard and tracer and a mouse monoclonal antibody. The intra- and inter-assay coefficients of variation are less than 10%. The sensitivity of the IGF-2 assay is 50 pg/ml.

### Immortalization of WT and PRLR KO preadipocytes

Brown adipocytes and their precursor cells were isolated from newborn mice by collagenase (1 mg/ml) digestion (Serva, Heidelberg, Germany) for 15 min at 37°C as described in [47]. Preadipocytes were thereafter immortalized by stable transfection with 2.5 µg of HA-TAg SV40 plasmid and 0.5 µg of pEGFP plasmid (Clontech, Palo Alto, CA) using Lipofectamin Reagent Plus (InVitrogen). The plasmid HA-TAg SV40, encoded the Large T Antigen of the simian virus 40 placed under the control of the proximal promoter of the human mineralocorticoid receptor gene. This plasmid was originally used as a transgene and allowed to generate the T37i cell line derived from a hibernoma of a transgenic mouse [20]. The pEGFP plasmid encoded for the enhanced green fluorescent protein and for the NeoR gene. Transfected cells were selected in the presence of 400 µg/ml of geneticin (G418, InVitrogen). Selective medium was changed 3 times a week for 2 weeks. Foci of cells were isolated using cloning rings and expanded when possible. PRLR-rescued brown adipocytes were obtained by transfecting PRLR KO cells with a 2.2 kb mouse PRLR cDNA previously subcloned into pcDNA 3.1 hygro expression vector (InVitrogen) after selection with 200 µg/ml hygromycin.

### Cell culture

All established cell lines but also T37i brown adipose cell line were cultured in a complete medium composed of DMEM/HAM's F12, 10% fetal calf serum, 2 mM glutamine, 100 IU/ml penicillin, 100 µg/ml streptomycin, and 20 mM HEPES and were grown at 37°C in a humidified atmosphere with 5% CO_2_. Differentiation into mature brown adipocytes was achieved by incubating subconfluent cells with complete medium supplemented with 2 nM triiodothyronine (Sigma, Saint Quentin Fallavier, France) and 20 nM insulin for 7 days. All products were purchased from InVitrogen except when stated. Before each hormonal treatment, human PRL was produced by recombinant technology then purified as described previously [48]. Human IGF-2 was purchased from Calbiochem (La Jolla, CA).

### Immunodetection of the SV40 large T antigen

Immunodetection was performed using a monoclonal anti-SV40 large T Antigen antibody (Ab-2; Calbiochem). Cells were seeded on glass slides and fixed with 100% ethanol, rinsed with PBS, and incubated with a 1∶100 dilution of the primary antibody for 45 min at 37°C. After washing, cells were incubated for 30 min at 37°C with a 1∶150 dilution of the biotinylated goat anti-mouse antibody (Tebu, Le Perray en Yvelines, France) followed with an incubation for 30 min at 37°C with a 1∶100 dilution of CY3-streptavidine (Jackson ImmunoResearch Laboratories, West Grove, PA).

### Studies of the activation of STAT5 signaling pathway

Differentiated T37i cells, starved for 18 h in minimum medium, were stimulated with 100 ng/ml PRL, during 0, 5 and 15 min; cells were washed twice with ice-cold PBS and lysed as previously described [18]. Five hundred µg of total protein were immunoprecipitated overnight at 4°C with protein A sepharose (Amersham) and the rabbit polyclonal anti-STAT5 (C17 Santa Cruz), reduced in Laemmli buffer and a 8% acrylamide SDS-PAGE was performed. Electrotransferred proteins were incubated with monoclonal anti-phosphotyrosine (1/5000 clone 4G10 Upstate Biotechnology) followed by incubation with the secondary antibody: anti-mouse IgG peroxydase-linked (GE Healthcare), detected with ECL western blotting detection reagent (GE Healthcare) and exposed to a film (Konica X-ray film AX, Roissy, France). Blots were stripped by a 30 min-incubation at 50°C in 62.5 mM Tris-HCl pH 6.8, 2% SDS, 100 mM 2-mercaptoethanol, washed with TBS/0.1% Tween 20 and were incubated with anti-STAT5 and anti-rabbit IgG peroxydase-linked (GE Healthcare) as secondary antibody.

### EMSA

Cells were washed with cold PBS and homogenized in 20 mM HEPES, pH 7.9, 1.5 mM MgCl_2_, 600 mM NaCl, 0.2 mM EDTA, 0.5 mM phenylmethylsulfonyl fluoride, 25% glycerol and 0.1% protease and phosphatase inhibitor cocktail (Sigma) by 20 strokes in a glass-glass Potter apparatus at 4°C. The homogenates were centrifuged at 20,000×g for 30 min at 4°C, and the supernatant was used as whole cell extracts. Gel mobility shift assays were performed essentially as described in [49]. Purified oligonucleotides were annealed and labeled with [^32^P]-dCTP (GE Healthcare) using the Klenow fragment of DNA polymerase (Life Technologies, Inc., Paisley, UK) to a specific activity of approximately 10^8^ cpm/µg of DNA. Unlabeled oligonucleotides were used as competitors. Oligonucleotide sequences were as follows: IGF-2-forward: 5′-GCCAGATGTTCTTGGAATGGGACA-3′; IGF-2-rev: 5′-GTGTCCCATTCCAAGAACATCTGG-3′. Protein-DNA complexes were separated from free DNA by electrophoresis on nondenaturing 4.5% polyacrylamide gel in 0.25X Tris-borate-EDTA buffer at 200 V for 1 h. Gels were dried and exposed to x-ray film at −80°C.

### Proliferation assays

T37i cells were cultured in 24-well plates. Post-confluent cells (d2) and differentiated cells (d7) were rinsed and grown for 12 h in a serum-free medium (DMEM/HAM's F12 containing 2 nM T3), then maintained at 37°C in the same medium for an additional 8 h period, in the absence or presence of 20 nM IGF-2 or insulin, and 1 µCi/well of [^3^H]-Thymidine (GE Healthcare) was added during the last 2 h. Cells were washed twice with ice-cold PBS and lysed in 1% SDS. DNA was precipitated by 10% trichloroacetic acid. The resulting trichloroacetic acid-insoluble pellet was washed two times with 5% trichloroacetic acid and collected on Whatman GF/C filters. [^3^H]-Thymidine incorporation into DNA was measured by scintillation counting.

### Statistical analyses

All data were prepared for analysis with standard spread sheet software (Microsoft Excel). Data are expressed as the mean±SE. Statistical analysis was performed using Student's t test to determine significant differences among groups and were performed using the computer software Prism 4 (GraphPad Software, San Diego, CA). We performed ANOVA, if statistical significance (P values *<0.05, **<0.01 and ***<0.001) was achieved and post-test analysis to account for multiple comparisons was done.

## Supporting Information

Figure S1 Lipogenic capacity of brown adipocytes is not impaired in PRLR KO mice. A) Citrate synthase activity (CS) and B) Glycerol-3-phosphate dehydrogenase (G3PDH) were determined in homogenates isolated from the BAT of WT and KO mice. Results represent mean±SE of 6 animals. NS: not significant
(0.18 MB TIF)

Figure S2 COX activity nor COX2 DNA content are altered in PRLR KO mice. A) Mitochondrial COX2 DNA content was determined by quantitative real time PCR in homogenates isolated from the BAT of WT and KO mice. Results, expressed as attomol of COX2/fmol of 18S, represent mean±SE of 6 animals. NS: not significant B) Cytochrome c oxidase acitivity (COX, Fig. S2A) was determined in homogenates isolated from the BAT of WT and KO mice. Results represent mean±SE of 6 animals. NS: not significant.
(0.17 MB TIF)

Figure S3 Differentiated T37i brown adipocytes expressed IGF-1R, IGF-2R and IR-A. RT-PCR analyses of IGF-1 receptor (IGF-1R), mannose-6 phosphate receptor (IGF-2R), and Insulin receptor form A (IR-A) in fully differentiated T37i cells in the presence (+) or the absence (-) of the reverse transcriptase. Oligonucleotides were as followed: IGF-1R-forward 5′-CGGTGACTTCTGCTCAAATGC-3′; IGF-1R-reverse 5′-GAATGGCGGATCTTCACGTAG-3′. IGF-2R-forward 5′-CGAGGCCGAAACTCAGATAGA-3′; IGF-2R-reverse 5′-AAAACGGATGATGAATGCTGTG-3′; IR-A-forward 5′-GCTGGACTGTGGTGGATATTGA-3′; IR-A-reverse 5′-TCAAGGGATCTTCGCTTTCG-3′.
(0.15 MB TIF)

Text S1 (0.03 MB DOC)

## References

[1] Goffin V, Binart N, Touraine P, Kelly PA (2002). Prolactin: the new biology of an old hormone.. Annu Rev Physiol.

[2] Bole-Feysot C, Goffin V, Edery M, Binart N, Kelly PA (1998). Prolactin and its receptor: actions, signal transduction pathways and phenotypes observed in prolactin receptor knockout mice.. Endocr Rev.

[3] LeBaron MJ, Ahonen TJ, Nevalainen MT, Rui H (2007). In vivo response-based identification of direct hormone target cell populations using high-density tissue arrays.. Endocrinology.

[4] Freemark M, Kirk K, Pihoker C, Robertson MC, Shiu RC (1993). Pregnancy lactogens in the rat conceptus and fetus: circulating levels, distribution of binding, and expression of receptor messenger RNA.. Endocrinology.

[5] Royster M, Driscoll P, Kelly PA, Freemark M (1995). The prolactin receptor in the fetal rat: cellular localization of messenger RNA, immunoreactive protein, and ligand binding activity and induction of expression in late gestation.. Endocrinology.

[6] Freemark M, Driscoll P, Maaskant R, Petryk A, Kelly PA (1997). Ontogenesis of prolactin receptors in the human fetus in early gestation. Implications for tissue differentiation and development.. J Clin Invest.

[7] Cannon B, Nedergaard J (2004). Brown adipose tissue: function and physiological significance.. Physiol Rev.

[8] Ricquier D, Bouillaud F (2000). Mitochondrial uncoupling proteins: from mitochondria to the regulation of energy balance.. J Physiol.

[9] Handschin C, Spiegelman BM (2006). Peroxisome proliferator-activated receptor gamma coactivator 1 coactivators, energy homeostasis, and metabolism.. Endocr Rev.

[10] Rosen ED, Spiegelman BM (2000). Molecular regulation of adipogenesis.. Annu Rev Cell Dev Biol.

[11] Rosen ED, Hsu CH, Wang X, Sakai S, Freeman MW (2002). C/EBPalpha induces adipogenesis through PPARgamma: a unified pathway.. Genes Dev.

[12] Golozoubova V, Gullberg H, Matthias A, Cannon B, Vennstrom B (2004). Depressed thermogenesis but competent brown adipose tissue recruitment in mice devoid of all hormone-binding thyroid hormone receptors.. Mol Endocrinol.

[13] Jimenez M, Leger B, Canola K, Lehr L, Arboit P (2002). Beta(1)/beta(2)/beta(3)-adrenoceptor knockout mice are obese and cold-sensitive but have normal lipolytic responses to fasting.. FEBS Lett.

[14] Lehr L, Canola K, Asensio C, Jimenez M, Kuehne F (2006). The control of UCP1 is dissociated from that of PGC-1alpha or of mitochondriogenesis as revealed by a study using beta-less mouse brown adipocytes in culture.. FEBS Lett.

[15] Guerra C, Navarro P, Valverde AM, Arribas M, Bruning J (2001). Brown adipose tissue-specific insulin receptor knockout shows diabetic phenotype without insulin resistance.. J Clin Invest.

[16] Miki H, Yamauchi T, Suzuki R, Komeda K, Tsuchida A (2001). Essential role of insulin receptor substrate 1 (IRS-1) and IRS-2 in adipocyte differentiation.. Mol Cell Biol.

[17] Laustsen PG, Michael MD, Crute BE, Cohen SE, Ueki K (2002). Lipoatrophic diabetes in Irs1(-/-)/Irs3(-/-) double knockout mice.. Genes Dev.

[18] Viengchareun S, Bouzinba-Segard H, Laigneau JP, Zennaro MC, Kelly PA (2004). Prolactin potentiates insulin-stimulated leptin expression and release from differentiated brown adipocytes.. J Mol Endocrinol.

[19] Ormandy CJ, Camus A, Barra J, Damotte D, Lucas BK (1997). Null mutation of the prolactin receptor gene produces multiple reproductive defects in the mouse.. Genes Dev.

[20] Zennaro MC, Le Menuet D, Viengchareun S, Walker F, Ricquier D (1998). Hibernoma development in transgenic mice identifies brown adipose tissue as a novel target of aldosterone action.. J Clin Invest.

[21] Efstratiadis A (1998). Genetics of mouse growth.. Int J Dev Biol.

[22] Fowden AL, Ward JW, Wooding FP, Forhead AJ, Constancia M (2006). Programming placental nutrient transport capacity.. J Physiol.

[23] Collins S, Cao W, Robidoux J (2004). Learning new tricks from old dogs: beta-adrenergic receptors teach new lessons on firing up adipose tissue metabolism.. Mol Endocrinol.

[24] Brisken C, Ayyannan A, Nguyen C, Heineman A, Reinhardt F (2002). IGF-2 is a mediator of prolactin-induced morphogenesis in the breast.. Dev Cell.

[25] Frasca F, Pandini G, Scalia P, Sciacca L, Mineo R (1999). Insulin receptor isoform A, a newly recognized, high-affinity insulin-like growth factor II receptor in fetal and cancer cells.. Mol Cell Biol.

[26] Pandini G, Medico E, Conte E, Sciacca L, Vigneri R (2003). Differential gene expression induced by insulin and insulin-like growth factor-II through the insulin receptor isoform A.. J Biol Chem.

[27] Vasavada RC, Garcia-Ocana A, Zawalich WS, Sorenson RL, Dann P (2000). Targeted expression of placental lactogen in the beta cells of transgenic mice results in beta cell proliferation, islet mass augmentation, and hypoglycemia.. J Biol Chem.

[28] Freemark M, Avril I, Fleenor D, Driscoll P, Petro A (2002). Targeted deletion of the prolactin receptor: effects on islet development, insulin production, and glucose tolerance.. Endocrinology.

[29] Ben Jonathan N, Hugo ER, Brandebourg TD, Lapensee CR (2006). Focus on prolactin as a metabolic hormone.. Trends Endocrinol Metab.

[30] Ling C, Hellgren G, Gebre-Medhin M, Dillner K, Wennbo H (2000). Prolactin (PRL) receptor gene expression in mouse adipose tissue: increases during lactation and in PRL-transgenic mice.. Endocrinology.

[31] Imai T, Takakuwa R, Marchand S, Dentz E, Bornert JM (2004). Peroxisome proliferator-activated receptor gamma is required in mature white and brown adipocytes for their survival in the mouse.. Proc Natl Acad Sci U S A.

[32] Lin J, Wu PH, Tarr PT, Lindenberg KS, St Pierre J (2004). Defects in adaptive energy metabolism with CNS-linked hyperactivity in PGC-1alpha null mice.. Cell.

[33] Gray SL, Nora ED, Backlund EC, Manieri M, Virtue S (2006). Decreased brown adipocyte recruitment and thermogenic capacity in mice with impaired PPAR{gamma} (P465L PPAR{gamma}) function.. Endocrinology.

[34] Pearce S, Budge H, Mostyn A, Genever E, Webb R (2005). Prolactin, the prolactin receptor and uncoupling protein abundance and function in adipose tissue during development in young sheep.. J Endocrinol.

[35] Kedzia C, Lacroix L, Ameur N, Ragot T, Kelly PA (2005). Medullary thyroid carcinoma arises in the absence of prolactin signaling.. Cancer Res.

[36] Nanbu-Wakao R, Fujitani Y, Masuho Y, Muramatu M, Wakao H (2000). Prolactin enhances CCAAT enhancer-binding protein-beta (C/EBP beta) and peroxisome proliferator-activated receptor gamma (PPAR gamma) messenger RNA expression and stimulates adipogenic conversion of NIH-3T3 cells.. Mol Endocrinol.

[37] Freemark M, Fleenor D, Driscoll P, Binart N, Kelly PA (2001). Body weight and fat deposition in prolactin receptor-deficient mice.. Endocrinology.

[38] Flint DJ, Binart N, Kopchick J, Kelly P (2003). Effects of growth hormone and prolactin on adipose tissue development and function.. Pituitary.

[39] Flint DJ, Binart N, Boumard S, Kopchick JJ, Kelly P (2006). Developmental aspects of adipose tissue in GH receptor and prolactin receptor gene disrupted mice: site-specific effects upon proliferation, differentiation and hormone sensitivity.. J Endocrinol.

[40] Cianfarani S, Liguori A, Boemi S, Maghnie M, Iughetti L (2005). Inaccuracy of insulin-like growth factor (IGF) binding protein (IGFBP)-3 assessment in the diagnosis of growth hormone (GH) deficiency from childhood to young adulthood: association to low GH dependency of IGF-II and presence of circulating IGFBP-3 18-kilodalton fragment.. J Clin Endocrinol Metab.

[41] Tseng YH, Butte AJ, Kokkotou E, Yechoor VK, Taniguchi CM (2005). Prediction of preadipocyte differentiation by gene expression reveals role of insulin receptor substrates and necdin.. Nat Cell Biol.

[42] Liu J, DeYoung SM, Zhang M, Zhang M, Cheng A (2005). Changes in integrin expression during adipocyte differentiation.. Cell Metab.

[43] Kang S, Bajnok L, Longo KA, Petersen RK, Hansen JB (2005). Effects of Wnt signaling on brown adipocyte differentiation and metabolism mediated by PGC-1alpha.. Mol Cell Biol.

[44] Binart N, Helloco C, Ormandy CJ, Barra J, Clement-Lacroix P (2000). Rescue of preimplantatory egg development and embryo implantation in prolactin receptor-deficient mice after progesterone administration.. Endocrinology.

[45] Teixeira M, Viengchareun S, Butlen D, Ferreira C, Cluzeaud F (2006). Functional IsK/KvLQT1 potassium channel in a new corticosteroid-sensitive cell line derived from the inner ear.. J Biol Chem.

[46] Mohan S, Baylink DJ (1995). Development of a simple valid method for the complete removal of insulin-like growth factor (IGF)-binding proteins from IGFs in human serum and other biological fluids: comparison with acid-ethanol treatment and C18 Sep-Pak separation.. J Clin Endocrinol Metab.

[47] Klein J, Fasshauer M, Ito M, Lowell BB, Benito M (1999). beta(3)-adrenergic stimulation differentially inhibits insulin signaling and decreases insulin-induced glucose uptake in brown adipocytes.. J Biol Chem.

[48] Goffin V, Kinet S, Ferrag F, Binart N, Martial JA (1996). Antagonistic properties of human prolactin analogs that show paradoxical agonistic activity in the Nb2 bioassay.. J Biol Chem.

[49] Lombes M, Binart N, Oblin ME, Joulin V, Baulieu EE (1993). Characterization of the interaction of the human mineralocorticosteroid receptor with hormone response elements.. Biochem J.

