# Supplementary figures and images for: Prolactin Receptor Signaling Is Essential for Perinatal Brown Adipocyte Function: A Role for Insulin-like Growth Factor-2
Source: PLoS One. 2008 Feb 6;3(2):e1535. doi: 10.1371/journal.pone.0001535 (PMC2212135; doi:10.1371/journal.pone.0001535)

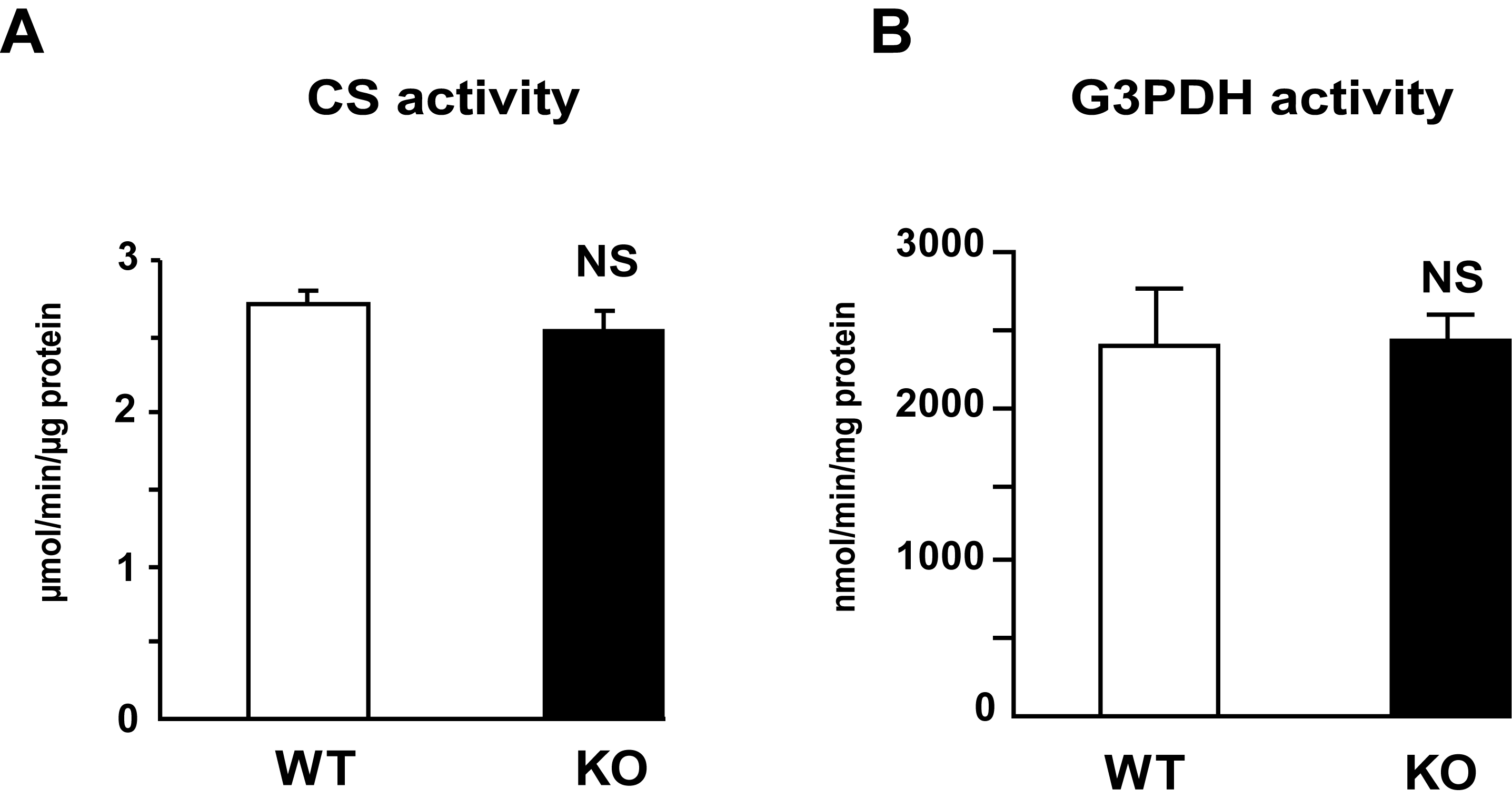

Supplement: Figure S1 — Lipogenic capacity of brown adipocytes is not impaired in PRLR KO mice. A) Citrate synthase activity (CS) and B) Glycerol-3-phosphate dehydrogenase (G3PDH) were determined in homogenates isolated from the BAT of WT and KO mice. Results represent mean±SE of 6 animals. NS: not significant (0.18 MB TIF) [file pone.0001535.s001.tif]

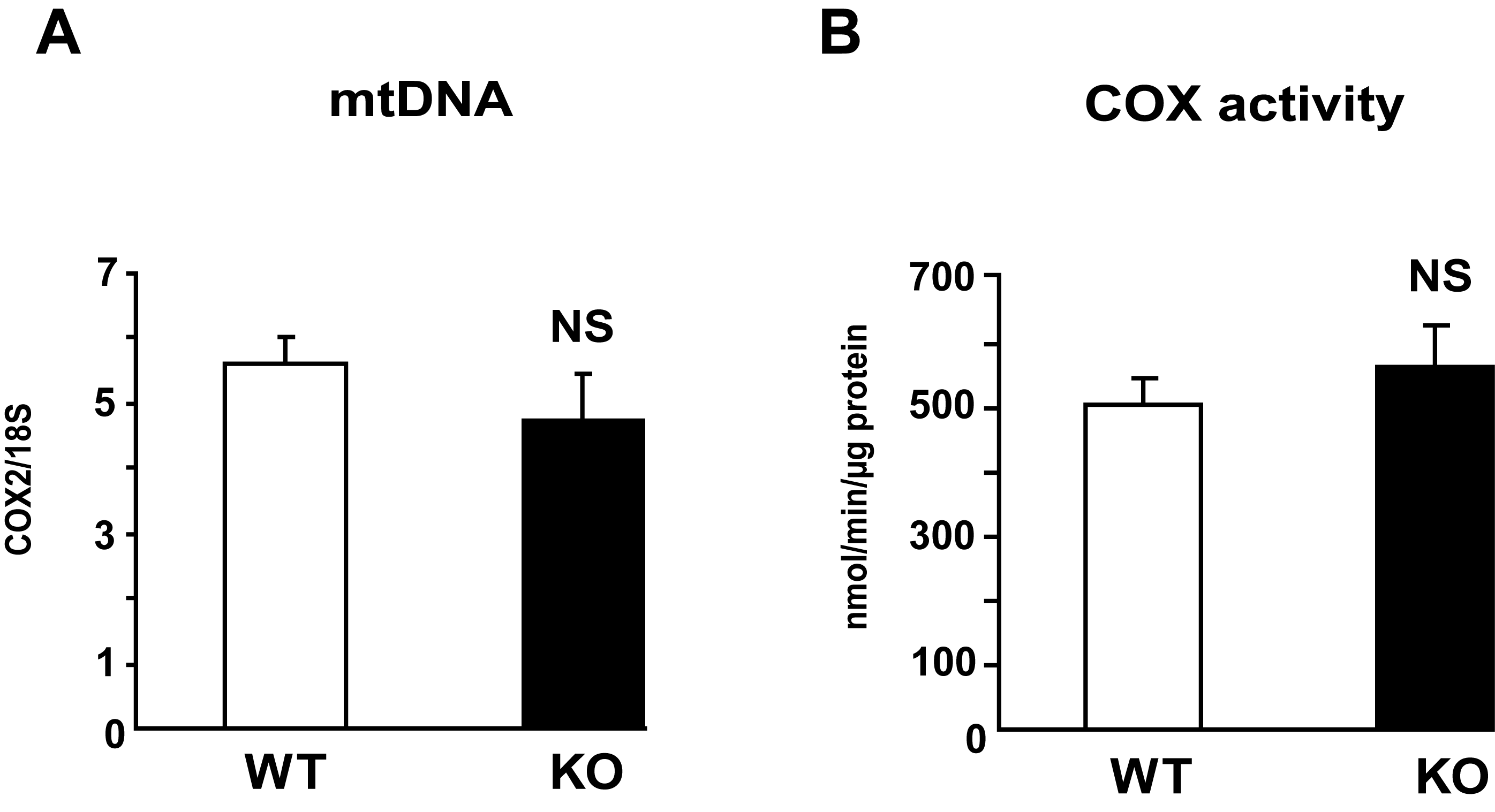

Supplement: Figure S2 — COX activity nor COX2 DNA content are altered in PRLR KO mice. A) Mitochondrial COX2 DNA content was determined by quantitative real time PCR in homogenates isolated from the BAT of WT and KO mice. Results, expressed as attomol of COX2/fmol of 18S, represent mean±SE of 6 animals. NS: not significant B) Cytochrome c oxidase acitivity (COX, Fig. S2A) was determined in homogenates isolated from the BAT of WT and KO mice. Results represent mean±SE of 6 animals. NS: not significant. (0.17 MB TIF) [file pone.0001535.s002.tif]

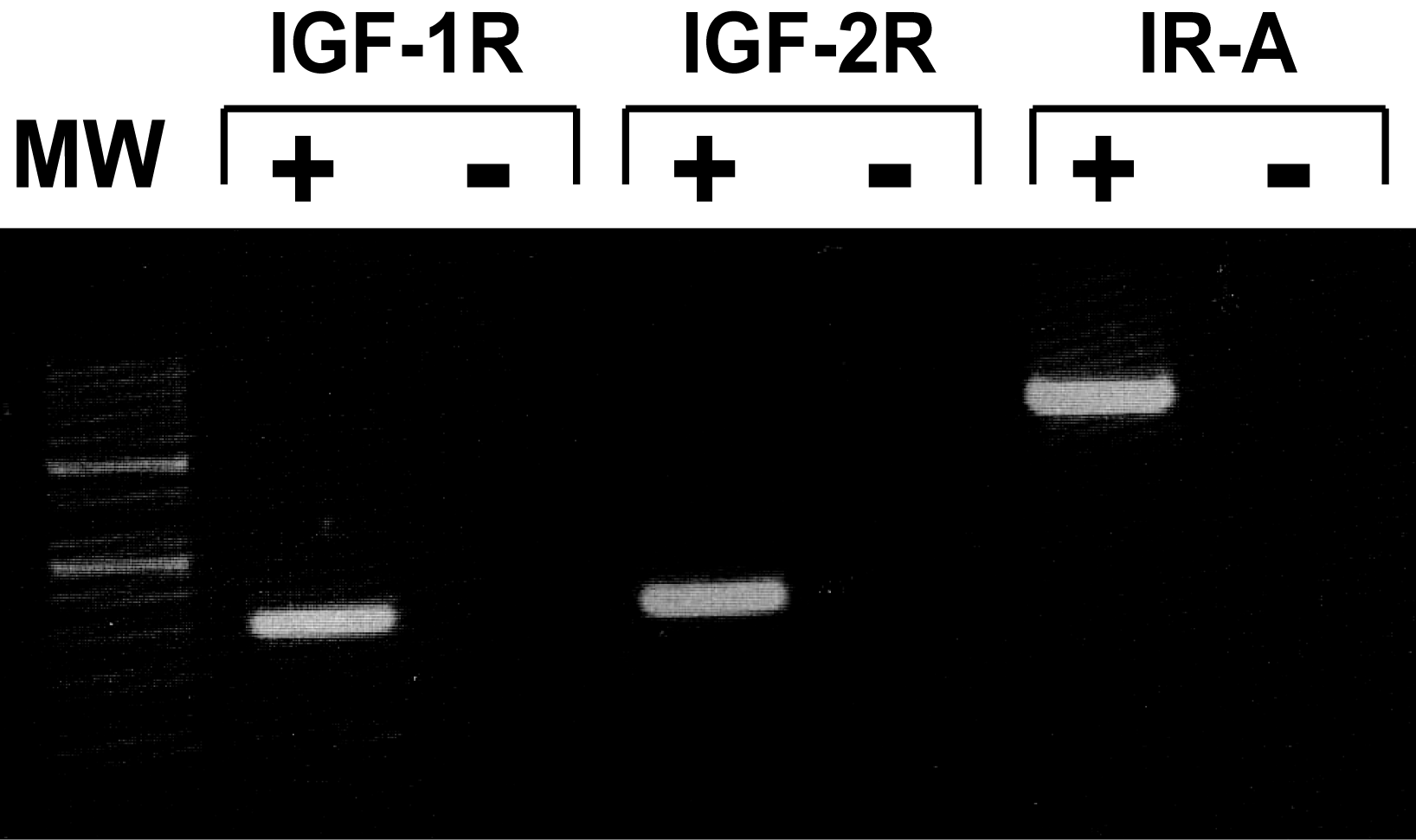

Supplement: Figure S3 — Differentiated T37i brown adipocytes expressed IGF-1R, IGF-2R and IR-A. RT-PCR analyses of IGF-1 receptor (IGF-1R), mannose-6 phosphate receptor (IGF-2R), and Insulin receptor form A (IR-A) in fully differentiated T37i cells in the presence (+) or the absence (-) of the reverse transcriptase. Oligonucleotides were as followed: IGF-1R-forward 5′-CGGTGACTTCTGCTCAAATGC-3′; IGF-1R-reverse 5′-GAATGGCGGATCTTCACGTAG-3′. IGF-2R-forward 5′-CGAGGCCGAAACTCAGATAGA-3′; IGF-2R-reverse 5′-AAAACGGATGATGAATGCTGTG-3′; IR-A-forward 5′-GCTGGACTGTGGTGGATATTGA-3′; IR-A-reverse 5′-TCAAGGGATCTTCGCTTTCG-3′. (0.15 MB TIF) [file pone.0001535.s003.tif]
