# Supplementary material for: Prolactin Receptor Signaling Is Essential for Perinatal Brown Adipocyte Function: A Role for Insulin-like Growth Factor-2
Source: PLoS One. 2008 Feb 6;3(2):e1535. doi: 10.1371/journal.pone.0001535 (PMC2212135; doi:10.1371/journal.pone.0001535)
Supplement: Text S1 — (0.03 MB DOC) [file pone.0001535.s004.doc]

##### Supporting Information

**Supplementary protocols**

**Cytochrome c oxidase and citrate synthase activities**

Spectrophotometric assays of cytochrome c oxidase (COX) and citrate synthase (CS) were performed in homogenates of BAT isolated from WT and KO mice according to [1,2].

**G3PDH Activity**

Glycerol-3-phosphate dehydrogenase (G3PDH) activity was assayed, in homogenates of BAT isolated from WT and KO mice, by recording the initial rate of oxidation of NADH at 340 nm at 25 °C [3].

**Mitochondrial COX2 Quantification**

Mitochondrial DNA quantification was performed on total DNA extracted from WT and KO BAT homogenates using standard procedures based on SDS-proteinase K digestion and isopropanol precipitation. Mitochondrial DNA (mtDNA) was quantified by real time PCR using COX2 gene on the mtDNA as target gene and, as standard, serial dilutions of linearized pGEMT-easy plasmid with the COX2 fragment, as insert. Oligonucleotides used for the amplification of COX2 were as followed: COX2-forward: 5'- GATAACCGAGTCGTTCTGCCA-3’; COX2-rev : 5'- CCCTGGTCGGTTTGATGTT
ACT -3'.

1. Wharton DC, Tzagoloff A (1967) Cytochrome oxidase from beef heart mitochondria. Methods Enzymol 10: 245-250.

2. Srere PA (1969) Citrate synthase. Methods Enzymol 13: 3-11.

3. Kozak LP, Jensen JT (1974) Genetic and developmental control of multiple forms of L-glycerol 3-phosphate dehydrogenase. J Biol Chem 249: 7775-7781.
